# Modified Peyton’s Technique in Pediatric Dental Education to Impart Knowledge and Clinical Skills to Dental Interns for the Preclinical Exercise of Stainless-Steel Crown Preparation: A Quasi-experimental Study

**DOI:** 10.7759/cureus.104041

**Published:** 2026-02-21

**Authors:** Tanvi Saraf, Farheen Tafti, Amit Patil, Shefali Talekar, Mrunmayee Soman, Anam Shaikh

**Affiliations:** 1 Pediatric and Preventive Dentistry, Bharati Vidyapeeth (Deemed to be University) Dental College and Hospital, Navi Mumbai, IND; 2 Endodontics, Bharati Vidyapeeth (Deemed to be University) Dental College and Hospital, Navi Mumbai, IND; 3 Conservative Dentistry and Endodontics, Bharati Vidyapeeth (Deemed to be University) Dental College and Hospital, Navi Mumbai, IND

**Keywords:** dental education, modified peyton’s approach, preclinical training, quasi-experimental study, stainless-steel crown

## Abstract

Background

Effective teaching of preclinical skills is crucial for dental interns’ transition to clinical practice. Conventional lectures lack the interactivity and reflection required for learning. The Modified Peyton’s four-step approach offers a structured, learner-centered method that promotes active engagement and skill retention.

Materials and methods

A quasi-experimental, double-blinded study was conducted among 70 dental interns randomly and equally assigned to Group A (Modified Peyton’s approach) or Group B (conventional technique). Knowledge was assessed using pre- and post-test multiple-choice questions (MCQs). Procedural skills were evaluated eight days later using a validated 25-item checklist by two blinded assessors and through self-assessment. Teaching conditions and materials were standardized, and examiners and statisticians were blinded.

Results

Both groups showed significant improvement (p < 0.001). Group A (n = 34) demonstrated greater knowledge gains (38.2%-94.1%) compared to Group B (n = 32) (75%-81.3%). Self-assessment gains for selected parameters were significantly higher in Group A (p < 0.001). Strong assessor-self-assessment agreement was observed in Group A, with greater discrepancies in Group B, indicating superior skill replication and self-evaluation consistency.

Conclusion

The Modified Peyton’s approach was more effective than didactic lectures in teaching stainless-steel crown preparation, resulting in improved knowledge, clinical skill performance, and self-assessment accuracy.

## Introduction

In dental education, preclinical skills are incorporated into the curriculum to allow for a smooth transition from preclinical to clinical training by improving hand-eye coordination [1]. The effectiveness of different teaching methods in skill laboratories remains a subject of debate in the literature. Traditionally, skill instruction involved the instructor demonstrating and explaining procedures, followed by students replicating what they observed. While this approach has been used for decades, it does not align with contemporary principles of adult learning [2].

The Peyton’s method was originally designed for a 1:1 teacher-to-student ratio but was adapted in 2014 for small group teaching, becoming known as the Modified Peyton’s method [3]. This structured, stepwise instructional strategy is considered practicable for tutors and well-accepted by trainees. Unlike the conventional, teacher-centred and passive instructional approach, modified Peyton’s technique emphasizes a student-centred paradigm, encouraging active participation and self-directed learning [4].

The stepwise sequence involves: (1) demonstration of the entire procedure at normal speed without interruption; (2) deconstruction, with the instructor explaining each sub-step in detail; (3) comprehension, where students guide the instructor through the procedure, promoting active recall; and (4) performance, where students independently carry out the procedure under supervision [5]. The comprehension phase in particular facilitates deeper understanding and retention, as learners reconstruct the procedural sequence.

Recent meta-analyses support Peyton’s method as an effective and student-centred approach for the development of procedural competencies in health professions education [6]. In dental education, small groups - typically five to eight trainees - receive training in a clinical skills laboratory. This study adapted and tested the teaching of pediatric stainless-steel crown preparation using the Modified Peyton’s method versus conventional methods, assessing outcomes in clinical skills and knowledge acquisition.

## Materials and methods

This methodology follows the Transparent Reporting of Evaluations with Nonrandomized Designs (TREND) checklist [7] to ensure structured and transparent reporting. The quasi-experimental study was conducted in 2025 at Bharati Vidyapeeth (Deemed to be University) Dental College and Hospital in Navi Mumbai, following approval from the Biomedical Ethics Committee (approval number: BEC480082024). All sessions took place in the institution’s clinical skills laboratory. Dental interns enrolled in the pediatric dental clinical posting during the study period who volunteered to participate were included, whereas those interns with prior formal training in stainless-steel crown preparation, interns unwilling to provide written informed consent, and those with temporary disabilities that prevented task performance were excluded.

Aim

The study aimed to assess the Modified Peyton’s technique in pediatric dental education as a means of imparting knowledge and clinical skills to dental interns performing stainless-steel crown preparation.

Objectives

The objectives of the study were: 1. To teach stainless-steel crown preparation via the Modified Peyton’s technique (Group A) and via conventional technique (Group B). 2. To assess and compare knowledge acquisition in both groups through pre- and post-intervention multiple-choice questions. 3. To assess skill acquisition using a standardized checklist via self-assessment across groups after eight days. 4. To compare checklist-based evaluations by Assessors 1 and 2 across both groups after eight days. 5. To assess skill acquisition by comparing self-assessment and assessor ratings within each group.

Sample-size determination

The sample size (n=70) reflected the number of interns who volunteered from the batch, following a pragmatic convenience approach. An intent-to-treat analysis was employed, including all consenting participants regardless of intervention completion. Formal a priori statistical power calculation was not conducted, given the exploratory nature and resource limitations. Future research should consider power analyses using effect size estimates from this study to guide sample-size planning. Interns were allocated to two groups of 35 each by a lottery system, ensuring unbiased distribution in an ecologically valid educational setting. Each group was subdivided into seven sub-groups of five interns each for intervention scheduling.

Interventions

Group A: Modified Peyton's Technique (MPT)

**Figure 1:**
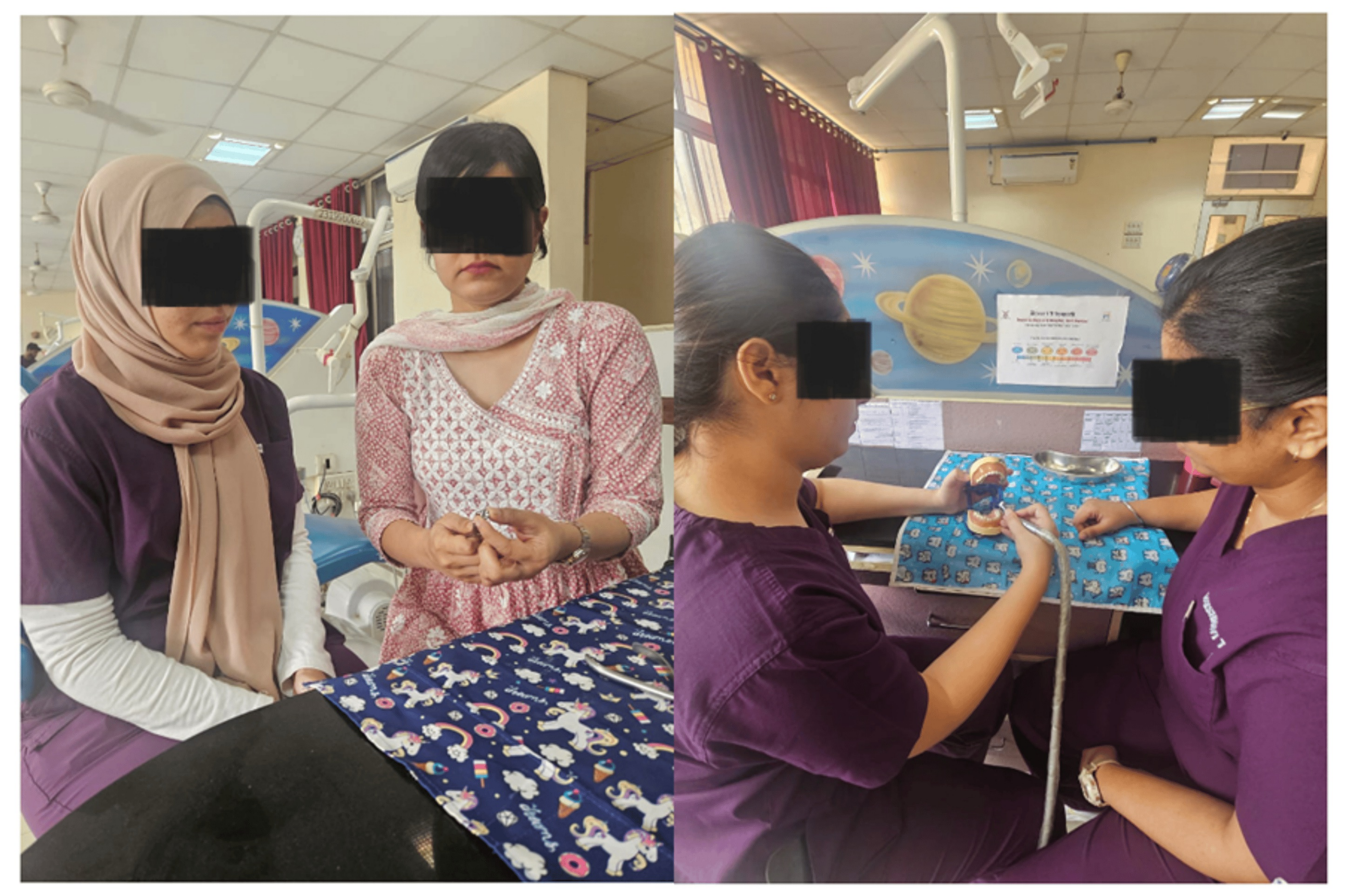
Modified Peyton's technique The primary investigator and an intern individually perform the steps of stainless-steel crown adaptation following the observing intern's instructions, until all interns of the group complete the stainless-steel crown adaptation exercise.

Each intern group received seven sessions (about half an hour each) over one week (Figure 1). The steps followed were (a) Demonstration: video demonstration of the procedure at normal speed without explanation; (b) Deconstruction: an interactive explanation with a video, deconstructing every aspect; (c) Comprehension: the primary investigator performed the steps of stainless-steel crown adaptation following the instructions of an intern while the other interns observed. The intern giving instructions was corrected by the primary investigator in the event of an error; (d) Performance of the procedure: the rest of the interns completed the exercise in the same manner [8].

Group B: Conventional Technique

**Figure 2:**
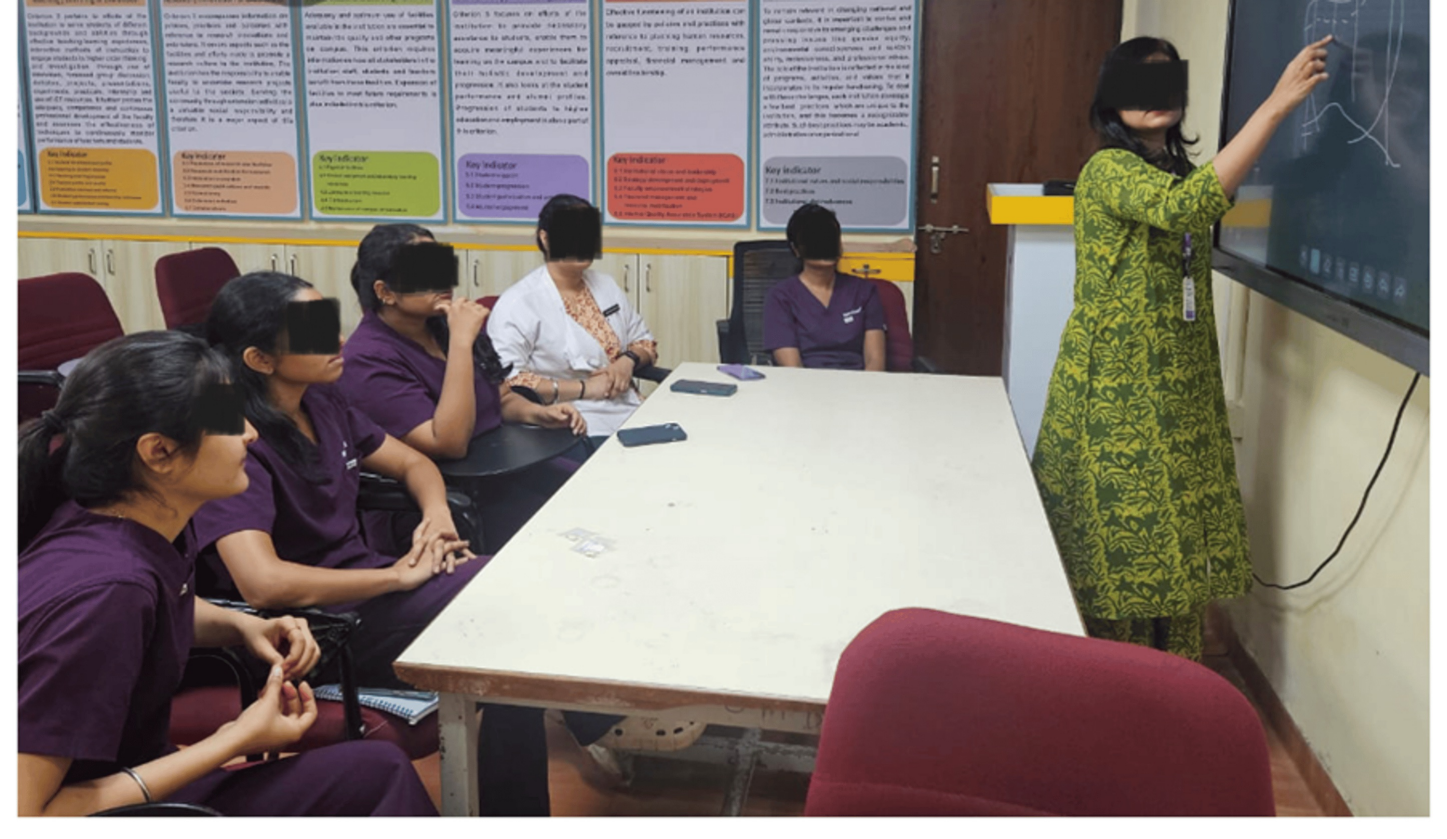
Conventional technique The primary investigator teaches the steps of stainless-steel crown adaptation through a lecture to a small group of interns.

The primary investigator delivered seven standardized sessions (about half an hour each) over one week, comprising an interactive lecture with a demonstration, followed by viewing procedural videos and clarifying doubts (Figure 2). All teaching sessions were delivered by the same instructor in the same environment at the same time, ensuring consistent exposure time and minimizing external confounding factors such as instructor variability. To promote attendance, reminders were sent to participants before each session.

Pre- and post-intervention assessment

To verify understanding of the procedural steps, the primary investigator developed a checklist with 25 questions that covered each step of stainless-steel crown preparation [9]. It was pre-validated before implementation to ensure content relevance and reliability, with a content validation index of 1.00, as this was based on standard guidelines [9]. The assessment of procedural skills included blinded evaluations by two faculty assessors, as well as self-assessment by the participating interns, to capture both objective and subjective learning outcomes (see tables in the Appendices). Both pre-test and post-test MCQ assessments used identical questions to measure knowledge retention, with the post-test administered on the eighth day after intervention. The primary investigator was blinded to assessment outcomes and was only involved in the teaching sessions. Assessors were blinded to group allocations and teaching methods. The intervention phase (self-assessment period) spanned eight consecutive days. The assessor evaluations occurred during the subsequent week.

**Figure 3:**
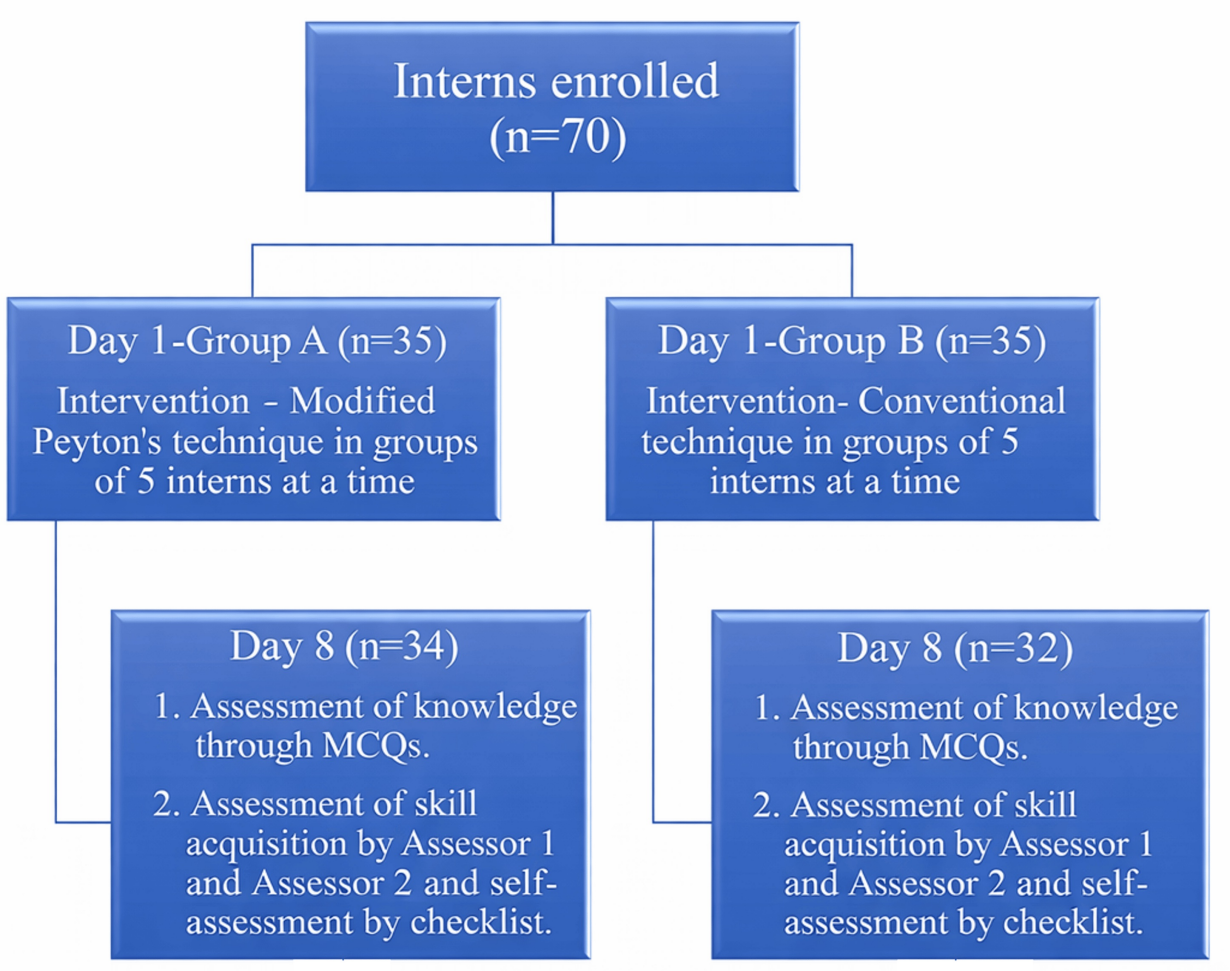
Flowchart of study design MCQs: multiple choice questions

Following data collection, all data were entered into a Microsoft Excel worksheet (Microsoft Corporation, Redmond, USA) and subsequently analyzed using IBM Statistical Package for Social Sciences (SPSS) software (IBM Corp., Armonk, USA). Categorical data were described in terms of frequencies and percentages. For interferential statistics of pre- and post-test comparison, McNemar’s test was used, whereas for categorical variables, the Chi-square test was used to compare proportions between groups; however, when expected cell frequencies were less than five, Fisher’s exact test was applied to ensure the accuracy of statistical inference. For all analyses, a p-value less than 0.05 was considered statistically significant.

## Results

Correct responses in Group A (n = 34) showed substantial improvement across all questions. The most notable changes were observed in Question 1 (0% to 94.1%), Question 2 (38.2% to 88.2%), Question 3 (8.8% to 91.2%), Question 4 (5.9% to 79.4%), Question 5 (14.7% to 91.2%), and Question 6 (2.9% to 91.2%), all statistically significant (p < 0.001) (Table 1).

**Table 1:** Comparison of responses in Group A (n=34), pre- and post-test The statistical test used is McNemar's test.

Question responses		Pre-test		Post-test		Percentage change		McNemar’s test χ² value	p value
		n	%	n	%				
						n	%		
Q1	Right	0	0%	32	94.10	32	94.10	56.726	<0.001
	Wrong	34	100%	2	5.90				
Q2	Right	13	38.20%	30	88.20	17	50	16.193	<0.001
	Wrong	21	61.80%	4	11.80				
Q3	Right	3	8.80%	31	91.20	28	82.40	42.882	<0.001
	Wrong	31	91.20%	3	8.80				
Q4	Right	2	5.90%	27	79.40	25	73.50	34.631	<0.001
	Wrong	32	94.10%	7	20.60				
Q5	Right	5	14.70%	31	91.20	26	76.50	36.892	<0.001
	Wrong	29	85.30%	3	8.80				
Q6	Right	1	2.90%	31	91.20	30	88.20	49.642	<0.001
	Wrong	33	97.10%	3	8.80				

Correct responses in Group B (n = 32) also showed substantial improvement across all questions. The most notable changes were observed in Question 1 (9.4% to 75%), Question 2 (9.4% to 62.5%), Question 3 (15.6% to 81.3%), Question 4 (6.3% to 62.5%), Question 5 (6.3% to 56.3%), and Question 6 (0% to 68.8%), all statistically significant (p < 0.001) (Table 2).

**Table 2:** Comparison of responses in Group B (n=32) pre- and post-test The statistical test used was McNemar's test.

Question responses		Pre-test		Post-test		Percentage change		McNemar’s test χ² value	p value
		n	%	n	%				
						n	%		
Q1	Right	3	9.40	24	75	21	65.60	25.626	<0.001
	Wrong	29	90.60	8	25				
Q2	Right	3	9.40	20	62.50	17	53.10	17.374	<0.001
	Wrong	29	90.60	12	37.50				
Q3	Right	5	15.60	26	81.30	21	65.60	25.024	<0.001
	Wrong	27	84.40	6	18.80				
Q4	Right	2	6.30	20	62.50	18	56.30	20.017	<0.001
	Wrong	30	93.80	12	37.50				
Q5	Right	2	6.30	18	56.30	16	50	16.364	<0.001
	Wrong	30	93.80	14	43.80				
Q6	Right	0	0	22	68.80	22	68.80	30.545	<0.001
	Wrong	32	100	10	31.30				

Comparison of the percentage change of correct responses between Group A and Group B in self-assessment showed improvement across all questions. Changes observed in Question 1 (94.1% in Group A and 65.6% in Group B) and Question 5 (76.5% in Group A and 50% in Group B ) were statistically significant (p < 0.001). However changes in Question 2 (50% in Group A and 53% in Group B), Question 3 (82.4% in Group A and 65.6% in Group B), Question 4 (73.5% in Group A and 56.3% in Group B), and Question 6 (88.2% in Group A and 68.8% in Group B) were not statistically significant (Table 3).

**Table 3:** Comparison of percentage change between Group A and Group B The statistical test used was the Chi-square test.

Question	Group A		Group B		Chi-square test χ² value	p value
	n	%	n	%		
Q1	32	94.10	21	65.60	6.755	0.009
Q2	17	50	17	53.10	0.8	1
Q3	28	82.40	21	65.60	2.412	0.12
Q4	25	73.50	18	56.30	3.033	0.081
Q5	26	76.50	16	50	9.672	0.001
Q6	30	88.20	22	68.80	2.67	0.102

On comparison of responses of self-assessment, Assessor 1, and Assessor 2 in Group A, the majority of questions showed uniform responses across all three evaluators. Changes observed in Questions 11 and 12 were minor (No response was 100% in self-assessment, 85.3% under Assessor 1 and Assessor 2; Yes response was 0% in self-assessment, 14.7% under Assessor 1 and Assessor 2). In Question 14, a small variation was observed (No response was 100% in self-assessment, 91.2% under Assessor 1 and Assessor 2; Yes response was 0% in self-assessment, 8.8% under Assessor 1 and Assessor 2). Neither change was statistically significant (Table 4).

**Table 4:** Comparison of responses on self-assessment, Assessor 1, and Assessor 2 in Group A The statistical test used was Fischer's exact test.

Question	Yes/No	n	Self-assessment (%)	n	Assessor 1 (%)	n	Assessor 2 (%)	Fisher's exact test χ² value	p value
										Q11	No	34	100	29	85.3	29	85.3	3.576	0.167
											Yes	0	0	5	14.7	5	14.7		
Q12	No	34	100	29	85.3	29	85.3	3.576	0.167
	Yes	0	0	5	14.7	5	14.7		
Q14	No	34	100	31	91.2	31	91.2	1.461	0.481
	Yes	0	0	3	8.8	3	8.8		

On comparison of the responses of self-assessment, Assessor 1, and Assessor 2 in Group B, the majority of questions showed uniform responses across all three evaluators, with statistically significant changes seen in Questions 3, 11, and 12; however, the change seen in Question 17 was not statistically significant. In Question 3, a small variation was observed (No response was 100% in self-assessment, 59.4% under Assessor 1 and Assessor 2; Yes response was 0% in self-assessment, 40.6% under Assessor 1 and Assessor 2). Changes observed in Question 11 were considerable (No response was 100% in self-assessment, 31.3% under Assessor 1 and Assessor 2; Yes response was 0% in self-assessment, 68.8% under Assessor 1 and Assessor 2). Changes observed in Question 12 were considerable (No response was 100% in self-assessment, 25% under Assessor 1 and Assessor 2; Yes response was 0% in self-assessment, 75% under Assessor 1 and Assessor 2). A slight change was also observed in Question 17 (No response was 40.6% in self-assessment, 31.3% under Assessor 1 and Assessor 2; Yes response was 59.4% in self-assessment, 68.8% under Assessor 1 and Assessor 2) (Table 5).

**Table 5:** Comparison of responses on self-assessment, Assessor 1, and Assessor 2 in Group B The statistical test used was Fisher's exact test.

Question	Yes/No	n	Self-assessment (%)	n	Assessor 1 (%)	n	Assessor 2 (%)	Fisher's exact test χ² value	p value
										Q3	No	32	100	19	59.4	19	59.4	15.204	<0.001
											Yes	0	0	13	40.6	13	40.6		
Q11	No	32	100	10	31.3	10	31.3	37.017	<0.001
	Yes	0	0	22	68.8	22	68.8		
Q12	No	32	100	8	25	8	25	44.094	<0.001
	Yes	0	0	24	75	24	75		
Q17	No	13	40.6	10	31.3	10	31.3	0.381	0.826
	Yes	19	59.4	22	68.8	22	68.8		

On comparison of responses from self-assessment between Group A and B, the majority of questions showed identical responses across all three evaluators, with differences in Questions 9, 10, and 17 being statistically significant. For Questions 9 and 10, the Yes response was 100% in Group A and 59.4% in Group B. For Question 17, the Yes response was 40.6% in Group A and 59.4% in Group B (Table 6).

**Table 6:** Comparison of responses from self-assessment between Group A and Group B The statistical test used was Fisher's exact test. ^**^Statistically significant.

Question	Yes/No	n	Group A (%)	n	Group B (%)	Fisher's exact test χ² value	p value
								Q9	No	34	100	3	9.4	51.345	<0.001**
									Yes	0	0	29	90.6		
Q10	No	34	100	3	9.4	51.345	<0.001**
	Yes	0	0	29	90.6		
Q17	No	34	100	13	40.6	28.348	<0.001**
	Yes	0	0	19	59.4		

On comparison of responses from Assessor 1 between Groups A and B, the majority of questions showed identical responses across all three evaluators, with differences in Questions 9, 10, 11, and 17 being statistically significant; however, differences observed in Question 14 were not statistically significant. For Questions 9 and 10, the Yes response was 0% in Group A and 90.6% in Group B, and the No response was 100% in Group A and 9.4% in Group B. For Question 11, the Yes response was 14.7% in Group A and 68.8% in Group B, and the No response was 85.3% in Group A and 31.3% in Group B. For Question 14, the Yes response was 88.8% in Group A and 0% in Group B, and the No response was 91.2% in Group A and 100% in Group B. For Question 17, the Yes response was 0% in Group A and 68.8% in Group B, and the No response was 100% in Group A and 31.3% in Group B (Table 7).

**Table 7:** Comparison of responses from Assessor 1 between Group A and Group B The statistical test used was Fisher's exact test.

Question	Yes/No	n	Group A (%)	n	Group B (%)	Fisher's exact testχ² value	p value
								Q9	No	34	100	3	9.4	51.345	<0.001
									Yes	0	0	29	90.6		
Q10	No	34	100	3	9.4	51.345	<0.001
	Yes	0	0	29	90.6		
Q11	No	29	85.3	10	31.3	17.745	<0.001
	Yes	5	14.7	22	68.8		
Q14	No	31	91.2	32	100	1.274	0.259
	Yes	3	88.8	0	0		
Q17	No	34	100	10	31.3	32.037	<0.001
								Yes	0	0	22	68.8

On comparison of responses from Assessor 2 between Groups A and B, the majority of questions showed identical responses across all three evaluators, with differences in Questions 3, 9, 10, 11, 12, and 17 being statistically significant; however, differences observed in Question 14 were not statistically significant. For Question 3, the Yes response was 100% in Group A and 30.6% in Group B, and the No response was 0% in Group A and 59.4% in Group B. For Questions 9 and 10, the Yes response was 0% in Group A and 90.6% in Group B, and the No response was 100% in Group A and 9.4% in Group B. For Questions 11 and 12, the Yes response was 14.7% in Group A and 68.8% in Group B, and the No response was 85.3% in Group A and 31.3% in Group B. For Question 14, the Yes response was 8.8% in Group A and 0% in Group B, and the No response was 91.2% in Group A and 100% in Group B. For Question 17, the Yes response was 0% in Group A and 68.8% in Group B, and the No response was 100% in Group A and 31.3% in Group B (Table 8).

**Table 8:** Comparison of responses from Assessor 2 between Group A and Group B The statistical test used was Fisher's exact test.

Question	Yes/No	n	Group A (%)	n	Group B (%)	Fisher's exact test χ² value	p value
								Q3	No	34	0	19	59.4	25.526	<0.001
Yes	0	100	13	30.6											
Q9	No	34	100	3	9.4	51.345	<0.001
	Yes	0	0	29	90.6		
Q10	No	34	100	3	9.4	51.345	<0.001
	Yes	0	0	29	90.6		
Q11	No	29	85.3	10	31.3	17.745	<0.001
	Yes	5	14.7	22	68.8		
Q12	No	29	85.3	8	25	21.943	<0.001
	Yes	5	14.7	24	75		
Q14	No	31	91.2	32	100	1.274,	0.259
	Yes	3	8.8	0	0		
Q17	No	34	100	10	31.3	32.037	<0.001
	Yes	0	0	22	68.8		

## Discussion

Peyton’s teaching approach conventionally consists of two steps: demonstration and practice. However, to enhance its applicability for group teaching, it was modified into a four-step strategy for small-group instruction. This modification is supported by Nourkami-Tutdibi (2020), who found that additional steps, while more time-consuming, significantly improved long-term retention of procedural knowledge [10].

Self-assessment and blinded assessor ratings indicated that, within Group A, nearly all evaluation questions showed uniform responses, with only minor, non-significant variations seen in questions** **11, 12, and 14. In contrast, Group B displayed statistically significant discrepancies on questions 3, 11, and 12 across self-assessment and faculty ratings, as well as notable variation in question 17, which did not reach significance. When comparing self-assessment between groups, the majority of responses were consistent, though significant differences emerged for questions 9, 10, and 17, favoring the Modified Peyton's group. Similarly, Assessor 1 and Assessor 2 ratings revealed significant intergroup differences for questions 9, 10, 11, and 17, with the conventional group consistently demonstrating higher rates of “Yes” responses in these domains.

These findings highlight that the Modified Peyton's approach led to more consistent and accurate skill replication and evaluation, particularly for steps with previously limited performance under conventional training. Overall, the results support the superior reliability and educational impact of the modified Peyton’s methodology in procedural skill teaching. Cooper and Tisdell (2020) [11] and Szulewski et al. (2020) [12] have suggested that Peyton’s technique reduces the cognitive load, which in turn increases the concentration, thus resulting in enhanced skill acquisition. The learner becomes an active participant and not a passive consumer in the demonstration of the skill set being demonstrated [13].

These findings align with prior research and systematic reviews, such as the meta-analysis by Giacomino et al. (2020) [6], which highlighted Peyton’s method as an effective, stepwise, interactive format promoting active engagement and cognitive reinforcement. Further, studies by Singhania and Pisulkar (2022) [1] Romero et al. (2018) [14], Sturm et al. (2023) [15], and Qutieshat (2018) [16] have demonstrated similar benefits in both medicine and dentistry, affirming its role in improving technical skills, decreasing procedural anxiety, and supporting learner confidence and comprehension.

In our study, the Modified Peyton’s technique proceeded smoothly without any major barriers or deviations from the planned protocol. The uniform teaching environment through the Modified Peyton’s technique, consistent instructor delivery, and scheduled session reminders contributed to high consistency in intervention administration. There was no discrepancy in the assessors, and the Cohen Kappa coefficient was 1.00. Although small-group teaching posed usual logistical challenges such as coordinating session timing and ensuring participant attendance, these were managed effectively through proactive communication with interns. No significant issues related to resource limitations, participant engagement, or instructional delivery were encountered, supporting the feasibility of this method in similar clinical education settings. The attrition was low (one in Group A and three in Group B), citing medical reasons for non-reporting to the institute on the scheduled day of assessment.

The primary strengths of the present study include the comprehensive assessment framework and minimized confounders through standardized teaching conditions. Nonetheless, several limitations warrant consideration. The modest sample size may limit the generalizability of the results beyond this setting. The study focused on short-term retention, so the durability of acquired skills remains uncertain, underscoring the need for future longitudinal assessments. Additionally, the potential influence of the Hawthorne effect remains, as participants’ awareness of being evaluated may have influenced performance [17]. However, this study demonstrates a novel teaching technique and needs further research.

In the context of competency-based dental education, the Modified Peyton's technique supports the development of reflective, skilled, and confident clinicians. Its phase-based instructional design - demonstration, deconstruction, comprehension, and performance - effectively bridges the gap between theory and practice, fostering deeper cognitive and psychomotor integration in procedural training.

## Conclusions

The Modified Peyton’s approach proved to be more effective than the conventional didactic lecture approach in teaching stainless-steel crown preparation to dental interns. It facilitated superior knowledge gain, skill execution, and self-assessment accuracy. Its structured, interactive, and feedback-oriented nature makes it an invaluable tool in dental pedagogy. Nevertheless, further longitudinal and multicentric research is warranted to confirm its long-term educational benefits and applicability across diverse clinical training scenarios.

## Appendices

**Table 9:** Multiple-choice questions

Question 1	How is occlusion checked?
Option A	With modelling wax
Option B	With occluding tooth
Option C	This is an optional step
Option D	Both a and b (CORRECT ANSWER)
Question 2	How is the stainless-steel crown size selected?
Option A	Preparing study model
Option B	Measuring mesiodistal width of tooth before preparation
Option C	Trial and error
Option D	All of the above (CORRECT ANSWER)
Question 3	After trimming, the stainless-steel crown is contoured by?
Option A	Walking the crimping plier
Option B	Adapting the contouring plier
Option C	Walking the contouring plier (CORRECT ANSWER)
Option D	This is an optional step
Question 4	The material of choice for cementation of stainless-steel crown is?
Option A	Type I Glass Ionomer cement CORRECT ANSWER)
Option B	Type II Glass Ionomer cement
Option C	Zinc phosphate cement
Option D	Zinc oxide eugenol cement
Question 5	Regarding the proximal reduction of the tooth in the preparation for a stainless steel crown, which sentence(s) is / are true:
Option A	Contact with the adjacent teeth must be broken gingivally and buccolingually and the adjacent tooth structure should not be damaged.
Option B	Proximal slices converge slightly toward the occlusal and lingual with no over-tapering.
Option C	The gingival margins should have a feather-edge finish line
Option D	All of the above are true.(CORRECT ANSWER)
Question 6	The most important principle(s) in the selection of the size of a stainless-steel crown is (are):
Option A	It should establish correct occluso gingival crown length.
Option B	The crown margins should follow the natural contours of the tooth’s marginal gingiva
Option C	The smallest crown that completely covers the preparation.
Option D	All of the above are true. (CORRECT ANSWER)

**Table 10:** Checklist for assessment

Question No.	Details	Option	Correct answer
1.	Was straight bur/flame shaped bur used for occlusal cutting? (Yes/No)	(Yes/No)	Yes
2.	Is the occlusal reduction approximately 1-1.5 mm with reference to the adjacent tooth?	(Yes/No)	Yes
3.	Was the occlusal surface flat after occlusal reduction?	(Yes/No)	No
4.	Was a taper fissure bur used for proximal cutting?	(Yes/No)	Yes
5.	Was contact with mesial tooth broken?	(Yes/No)	Yes
6.	Was contact with distal tooth broken?	(Yes/No)	Yes
7.	Was there a 2-to-4-degree convergence taper mesially?	(Yes/No)	Yes
8.	Was there a 2-to-4-degree convergence taper distally?	(Yes/No)	Yes
9.	Was buccal reduction done?	(Yes/No)	No
10.	Was lingual reduction done?	(Yes/No)	No
11.	Was adjacent tooth damaged mesially?	(Yes/No)	No
12.	Was adjacent tooth damaged distally?	(Yes/No)	No
13.	Was the gingival margin knife edged?	(Yes/No)	Yes
14.	Is there a ledge formation?	(Yes/No)	No
15.	Was there a snap fit?	(Yes/No)	Yes
16.	Was the stainless steel crown trimmed?	(Yes/No)	Yes
17.	Is occlusion maintained with occluding tooth?	(Yes/No)	No
18.	Was the contouring done with pliers on buccal?	(Yes/No)	Yes
19.	Was the contouring done with pliers on lingual?	(Yes/No)	Yes
20.	Was the contouring done with pliers on mesial?	(Yes/No)	Yes
21.	Was the contouring done with pliers on distal? (Yes/No)	(Yes/No)	Yes
22.	Was crimping done on buccal?	(Yes/No)	Yes
23.	Was crimping done on lingual?	(Yes/No)	Yes
24.	Was crimping done on mesial?	(Yes/No)	Yes
25.	Was crimping done on distal?	(Yes/No)	Yes
